# An Analysis of the Generalizability and Stability of the Halo Effect During the COVID-19 Pandemic Outbreak

**DOI:** 10.3389/fpsyg.2021.631871

**Published:** 2021-03-24

**Authors:** Giulio Gabrieli, Albert Lee, Peipei Setoh, Gianluca Esposito

**Affiliations:** ^1^Psychology Program, School of Social Sciences, Nanyang Technological University, Singapore, Singapore; ^2^LKC School of Medicine, School of Social Sciences, Nanyang Technological University, Singapore, Singapore; ^3^Department of Psychology and Cognitive Science, University of Trento, Trento, Italy

**Keywords:** halo effect, aesthetics, trustworthiness, SARS-nCoV-2, ethnicity

## Abstract

The influence on the global evaluation of a person based on the perception of a single trait is a phenomenon widely investigated in social psychology. Widely regarded as *Halo effect*, this phenomenon has been studied for more than 100 years now, and findings such as the relationship between aesthetic perception and other personality traits—such as competence and trustworthiness—have since been uncovered. Trustworthiness plays an especially crucial role in individuals' social interactions. Despite the large body of literature published on the Halo effect, and especially on the relationship between aesthetic appearance and perceived trustworthiness, little is known about the overall generalizability of the effect, as almost all of the studies have been conducted on adult participants from Western countries. Moreover, little is known about the stability of the effect over time, in the event of major destabilization, such as the outbreak of a pandemic. In this work, the cross-cultural generalizability of the *Halo effect* is investigated before and during the first few months of the COVID-19 pandemic. An analysis of the generalizability and stability over time of the *Halo effect* is presented. Participants (*N* = 380, *N* = 145 Asians, *N* = 235 Caucasians) have been asked to rate the aesthetic appearance and perceived trustworthiness of a set of human faces of different ages, gender, and ethnicity. Result of our analysis demonstrated that the *Halo effect* (Aesthetic × trustworthiness) is influenced by the age of presented faces, but not by their gender or ethnicity. Moreover, our results show that the strength of the effect can be affected by external events and that the volatility is higher for adults' than children's faces.

## 1. Introduction

The *Halo effect* (HE) is a cognitive bias in impression formation whereby the general evaluation of individuals' attributes is based on the evaluation of a single attribute (Nisbett and Wilson, 1977). When applied to aesthetic appearance, the HE is observed when the physical appearance is used as a basis for the evaluations of other attributes that are unrelated to appearance whatsoever. For example, a stranger who looks good is also perceived as intelligent or smart, even though intelligence and smarts are unrelated to physical attractiveness (Todorov et al., 2009). As a subclass of the confirmation bias in impression formation (Nickerson, 1998), the HE is known to be intuitive, pervasive, and constant (Cooper, 1981; Feldman, 1986; Kozlowski et al., 1986; Feeley, 2002). The HE is a widely investigated psychological phenomena, with an impact on different academic fields such as social psychology, computer science, and empirical aesthetics (Hartmann et al., 2008; Todorov et al., 2009; Tuch et al., 2012; Ferrari et al., 2017).

### 1.1. Aesthetics and Trustworthiness

The term “Halo Effect” was first proposed by Thorndike (1920) to describe the radiating effects of a single attribute on the evaluations of other attributes. The term resonates with paintings from the medieval period, in which saints were often crowned with a glowing circle around their heads, representing their general reverence or goodness. Empirically, the HE has been observed in numerous domains of impression formation. Early demonstrations of the effect (e.g., Asch, 1946), for instance, have shown that central attributes, such as social warmth or physical appearance, have predictable and radiating effects on the inferences of other attributes. Compared to an unattractive person, an attractive person is often assumed to be happier, more competent at work, more successful in marriage, even though none of these inferences are supported by evidence (Dion et al., 1972). Consistent with this work, other studies have demonstrated the HE of physical appearance in a host of social domains, from intellect (Landy and Sigall, 1974) and personality (Little et al., 2006) all the way to moral deservingness (Dion, 1972; Forgas et al., 1983), integrity (Dion, 1972), and many more (see Eagly et al., 1991, for a review).

Together, these results cast light on the associative nature of impression formation. That is, inferences about others are generally guided by the implicit rule that whatever good (e.g., beautiful) goes with the good (e.g., generous), and whatever bad (e.g., unattractive) goes with the bad (e.g., unintelligent). Such a rule, compatible with the Gestalt principle of coherence (Thagard, 2000), is regarded as a cognitive explanation for the HE. In the next paragraphs, we focus on how appearance may affect the perception of trustworthiness.

The impact of aesthetic appearance on perceived trustworthiness, also known as HE aesthetics × trustworthiness (Todorov et al., 2009), has been studied since the early years of the twentieth century. Unlike aesthetic appearance, trustworthiness is a global or “umbrella” trait that is fundamental to social perception (Fiske et al., 2007), with diverse implications in numerous life domains, such as in assessing another person's good or ill intentions.

Other works have replicated the impact of aesthetic appearance on perceived trustworthiness, with more aesthetically/physically attractive individuals being perceived as more trustworthy. For example, in a study conducted by Carter (1978) on the appearance of counselors—a replication of a previous study conducted by Cash et al. (1975)—revealed that attractive counselors are also perceived as more intelligent, warm, competent, and trustworthy. The strength of the effect was further confirmed in a review (Eagly et al., 1991) where aesthetic attractiveness was found to be positively linked with perceived social competence across 76 studies.

### 1.2. But Is Attractive Always Trustworthy?

Despite the large body of literature on the relationship between aesthetic appearance and trustworthiness, several questions remain unanswered. Almost all of the available literature focused, in fact, on adult individuals sampled from the WEIRD population, rendering generalizability an issue (Henrich et al., 2010; Jones, 2010). Moreover, even though some studies have been conducted on children's faces, demonstrating that the effect exists in children (Dion et al., 1972), there are limited comparisons on the impact of adults vs. children targets. As children's faces are known to be special stimuli that automatically capture adults' visual attention and elicit parental care (Brosch et al., 2007; Proverbio and De Gabriele, 2019; Venturoso et al., 2019), the HE may be present with different strengths between adult and child faces. Not only children's faces, but also adults' faces with facial traits that resemble the stereotypical traits of children, such as big round eyes, have been shown to influence adult viewers' estimations such that baby-faced adults are perceived as more trustworthy, warm, and innocent (see Zebrowitz, 1997, for a review). Moreover, repeated exposure to the same face has been reported to influence viewers' judgments of others' traits and skills, such as in the judgment of politicians' competence (Zajonc, 2001; Verhulst et al., 2010).

Finally, controversial results have been found for what concerns the importance of the rated individuals' gender. Significant differences between the scores given to males and females have been found in the works of Carter (1978), but not in others (Wetzel et al., 1981). One possibility for this is that in Carter (1978), there was an additional stereotype playing a part in the interaction participant gender × counselor gender), which is in people's mental representation of the stereotypical counselor (Chambers, 1983). To overcome the limitations of previous studies, this study aims to verify how the (a) ethnicity (ingroup vs. outgroup), (b) age (adult vs. baby), (c) gender (male vs. female), and (d) aesthetic attractiveness combine in shaping trust perception. More specifically, in this work, we investigate the aesthetics and trustworthiness perception of Asians and White/Caucasians adults raters of both adults' and children's faces, both males and females, of Asians and Caucasians ethnicities.

The data collection stage of the project, with the methods described in section 2.2, started in August 2019 and continued through April 2020. The data collection phase overlapped with the COVID-19 pandemic outbreak. Serendipitously, the data collected for this project allowed us to investigate the stability of the HE over time over time. One additional hypothesis—H_2_—was therefore added to study such effects.

### 1.3. Aim and Hypothesis

We formulated two hypotheses. The first hypothesis, analytic plan, and method were pre-registered on the Open Science Framework; the second hypothesis was formulated after beginning the data collection. The complete analytic plan is reported in section 2.3.

H_1_: “*Aesthetic attractiveness is positively correlated with perceived trust (HE). We predict the age of presented face to have an effect on the strength of the relationship, with the strength of the correlation higher for adults than for children's targets, but not the ethnicity or the gender of presented face.”*

**Rationale**: Children's faces elicit parental care regardless of kinship and hence, capture greater attention compared to adults faces (Brosch et al., 2007; Glocker et al., 2009; Parsons et al., 2011; Venturoso et al., 2019). Additionally, a recent study conducted by Collova et al. (2019)—based on a two-dimensional model (trustworthiness and dominance) from Oosterhof and Todorov (2008)—investigated whether children's faces elicit the same signal threat responses to adults' faces. Results of Collova's studies revealed that adults rate children's faces on different dimensions to adults' faces. More specifically, when rating children's faces, the evaluation is not based on trustworthiness. This suggests that evaluation of children's faces are not judged on their perception of trustworthiness, regardless of how aesthetically attractive they are. If so, one should expect the relationship between aesthetic appearance and trustworthiness to be stronger for adults' ratings of adults' faces as compared to children's faces. Therefore, we can expect the relationship between aesthetic appearance and trustworthiness to be stronger for adults' ratings of adults', as compared to adults' ratings of children's faces. From prior work, we know that gender (Wetzel et al., 1981) and ethnicity (Xu et al., 2012) do not seem to moderate the HE. But for the sake of completion, we decided to investigate these two demographic variables, with the expectation that neither gender nor ethnicity will have a significant impact on our observed results. In line with previous studies, we do not expect to find a significant impact of gender on the strength of the effect. With regard to ethnicity, differences may be present in the aesthetic ratings given to individuals of the ingroup or of the outgroup. However, as the implicit judgment of trustworthiness is based on the elaboration of facial cues that occur faster than the elaboration of ethnicity-specific traits (e.g., shape of the eyes; Engell et al., 2007), we do not expect any differences between the strength of the effect for ingroup and outgroup are expected.

H_2_: “*When individuals are asked to rate the aesthetic and trustworthiness of others' faces, we expect to see changes in the variability of the ratings after the diffusion of news about COVID-19 in trustworthiness but not aesthetic judgments toward adults but not children's faces.”*

**Rationale**: Research has established that Asian and Caucasian faces are perceived as distinct categories (Zhou et al., 2020). In a study conducted by Xu et al. (2012), it was reported that when making inferences about the trustworthiness of others from their aesthetic appearance, Chinese and Caucasians adopt the same strategies. However, Koopmans and Veit (2014) found that negative inter-ethnic contact can cause reduced trust toward members of the outgroup. In light of the COVID-19 pandemic global threat, following the diffusion of news about the spreading of the novel coronavirus in China, and with politicians targeting a specific ethnic group (Zheng et al., 2020), we can expect the situation to bias non-Asians against Asians, hence reducing Caucasians' estimation of trustworthiness, but not of aesthetics, toward Asian adults' faces. Previous research work by Fincher et al. (2008) highlighted that regions with a stronger history of contagious diseases are more likely to adopt collectivistic behaviors, including outgroup hostilities. It is therefore possible that, with the subsequent outbreak in Western countries, together with the adoption of specific measures to counter the diffusion of the virus in Eastern countries, collectivist beliefs brought about a reduction in the perceived trustworthiness, but not aesthetics, of Caucasians as evaluated by Asians. Such findings will suggest that salient threats of contagion, such as during the COVID-19 pandemic, may elicit the tendency to prefer interactions with familiar ingroups and reject unfamiliar outgroups. This tendency, given its strong evolutionary undertone, should be present in most people regardless of their culture. Account for this assumption, one should expect a global reduction of trust in the perception of adult faces, regardless of the cultural backgrounds of these adult faces. Such global reduction, however, should not be observed in the aesthetic perception, which unassociated with the threat of contagion. Taken together, these hypotheses suggest that we should see a generalized reduction of trust, but not aesthetics, toward both Asians and Caucasians adults' faces. For evaluation of children's faces, a different situation is expected. In an event-related potential (ERP) study conducted by Proverbio and De Gabriele (2019), it is reported that the other-race effect does not apply to infants' faces, supporting the specificity of the age of a face over its ethnicity for young faces. Differences in adults' perception of adults' and children's faces in other-race effects studies were also reported by Kuefner et al. (2008), in a series of three experimental studies. These findings suggest that the salience of infants' and children's faces should limit the impact of race on the estimation of other traits. Building on the work from Collova et al. (2019) reported above (H_1_), we can expect an early evaluation of infants' faces not to have an influence on perceived trustworthiness. Taken together, findings on the specificity of infants' and children's faces suggest that the age dimension plays a prominent role, more than the possible perceived threat dimension, in the evaluation of children's faces. It is therefore possible that, when presented with faces of children, adults' trustworthiness judgments are less likely to be influenced by the aesthetic traits of a child's face, as compared to when they are rating an adult's face. From a biological point of view, this behavior would reflect mammals', and especially humans', altruistic responses toward infants (Preston, 2013). Consequently, we do not expect any difference in the judgment of both the aesthetic and trustworthiness of children's faces before and during the initial stages of the COVID-19 pandemic outbreak.

## 2. Methods

### 2.1. Participants

The study was approved by the Internal Review Board of Nanyang Technological University (PSY-IRB-2019-008 and IRB-2019-10-019) and conducted according to the declaration of Helsinki. Informed consent was obtained from all the participants before the study. Participants (*N* = 380, M age = 25.0±8.49) voluntarily participated and were recruited through the Nanyang Technological University's School of Social Sciences Research Participation System or online through different social media platforms, including Facebook, Twitter, and the Subreddit community “samplesize,” with no geographical constraints. These social media and communities were selected in order to ensure our Caucasian sample would be composed of participants from different geographical areas, and especially North America and Europe. The gender and ethnicity of participants are reported in Table 1.

**Table 1:** Participants' demographic information.

**Ethnicity**	**Gender**	***N***	**Age**
Asian	Male	75	22.5 ± 1.83
	Female	70	21.0 ± 3.06
Caucasian	Male	80	29.0 ± 11.81
	Female	155	26.0 ± 8.99

### 2.2. Study Design

#### 2.2.1. Stimuli

Participants were presented with 64 faces of two different age groups (32 adults, 32 children), genders (32 males, females), and ethnicities (Asians, Caucasians). This structure allowed for the presentation of eight faces per combination of age, gender, and ethnicity (e.g., 8 adult Asian male faces). Front-facing images of faces (*N* = 64) were selected from the FFHQ Dataset (Karras et al., 2019), a dataset containing 70,000 high-quality (1,024 × 1,024) images published on Flickr2, an online photo management, and sharing tool, under different creative commons and public domain licenses (Creative Commons BY 2.0, Creative Commons BY-NC 2.0, Public Domain Mark 1.0, Public Domain CC0 1.0, or U.S. Government Works license). The dataset itself is released under the Creative Commons BY-NC-SA 4.0 license by NVIDIA Corporation and has been successfully used in previous publications (Karras et al., 2019; Kynkäänniemi et al., 2019; Wang et al., 2019; Zhao et al., 2020). Stimuli selection was conducted in such a way to create groups of eight (*N* = 8) faces for each possible combination of age, gender, and ethnicity. While values of aesthetics pleasantness were not available in the source dataset, images were selected with the aim to cover all the possible spectrum of values for aesthetics for each combination of age, gender, and ethnicity. More specifically, for each combination, four (*N* = 4) images were selected among those we expected would have obtained low (<50) values of aesthetics, and four (*N* = 4) we expected would have been rated high (>50) in aesthetics. The manipulation successfully worked, as values that covered the whole spectrum of possible ratings were obtained, and enough variance was achieved for the set of faces in both aesthetics and trustworthiness ratings, of which we expected four images to receive lower ratings in aesthetics and four to receive higher ratings in aesthetics. Selected faces were presented in random order, with no time constraints.

#### 2.2.2. Procedure

After having signed the informed consent, participants were instructed about the scope and procedure of the experiment, as well as the taxonomy employed in the study. Participants rated each face for aesthetic pleasantness (“*How much do you like this person?”*) and trustworthiness (“*How much do you trust this person?”*) on a 100-point sliding scale, anchored from 1 being “not at all” not to 100 = “extremely.” The effectiveness of the first question at measuring aesthetic pleasantness has been verified comparing our results with previous works that focused on the relationship between liking and trustworthiness. More details are reported in section 4.

### 2.3. Analytic Plan

The analytic plan was pre-registered on the Open Science Framework. Additional information can be found online on the Open Science Framework (https://osf.io/5cge3). A power analysis was conducted to estimate the number of participants required for this study (H_1_). Given that previous works have found the effect size for the HE of human faces to be of medium strength, to take into account a possible bias in published works (Collaboration, 2015; Camerer et al., 2018), we assumed a very weak effect size to estimate the required number of participants. Assuming six groups (children/adult, male/female, Asian/Caucasian), a very weak effect size (Cohen's d = 0.1), and to achieve a power of 0.95 at a 0.05 alpha value, a power analysis conducted in G*Power (Faul et al., 2007, 2009) revealed that *N* = 330 participants are required to perform an analysis of variance. The strength of the HE is measured as the Pearson's correlation between aesthetic and trustworthiness ratings. To test H_1_, a 2 × 2 × 2 analysis of variance was employed to control for the existence of significant effects of gender, age, and ethnicity on the strength of the HE, measured as the Persons' correlation between aesthetics and trustworthiness judgments. A *z*-test is employed as a *post-hoc* test to test whether the HE is stronger for adults than children faces. Additionally, a confirmatory analysis is conducted by means of a multiple linear regression analysis.

For what concerns the second hypothesis (H_2_), four Levene's tests for equality of variance have been conducted on aesthetics and trustworthiness, comparing the variance of data collected before and after the diffusion of news about the novel coronavirus, once for adults' faces and one for children's faces. As a threshold, we used February 1, 2020, which is, according to *Google Trend*2, the moment in which people started to show interest toward the SARS-CoV-2. In order for H_2_ to be verified, we expected significant differences in the variance of trustworthiness ratings toward adults' faces before and after our threshold date, but not for adults' faces aesthetics ratings, nor for both aesthetics and trustworthiness ratings toward children's faces. To take into account the multiple numbers of tests conducted, a correction for multiple tests using the Benjamini–Hochberg procedure, with a false discovery rate of 0.10, is employed.

## 3. Results

### 3.1. Effect of Ethnicity, Age, and Gender on the Strength of the Halo Effect

To evaluate the effects of ethnicity, age, and gender on the strength of the HE, measured as the Pearson's correlation between aesthetics (mean = 55.97 ± 19.81) and trustworthiness (mean = 53.83 ± 21.82), an analysis of variance has been conducted. Results of the analysis of variance revealed only a main effect of age (*F*-value = 9.753, *p*-value = 0.00194, $\eta_{p}^{2}$=0.03, Effect size f = 0.18, correlation among repeated measures = 0.503, achieved power = 1.0) but no main effect of gender or ethnicity, as well as no significant effects of the interaction between age and gender, or gender and ethnicity on the strength of the HE (aesthetics × trustworthiness). A significant interaction between face's age and ethnicity (ingroup vs. outgroup) is highlighted (*F*-value = 6.31, *p*-value = 0.0124), such that the differences in strength of the HE between Ingroup's Adults and Children faces (*t*-value = 3.98, uncorrected *p*-value= 7.11 · 10^-5^) are bigger than the differences between Outgroup's Adults and Children faces (*t*-value = 1.22, uncorrected *p*-value = 0.221). This may however be caused by the diffusion of news about the COVID-19 pandemic outbreak. In fact, by repeating the analysis only on a subset of data collected before the initial diffusion of information about the novel coronavirus (*N* = 179), the interaction between ethnicity (ingroup or outgroup) and age of presented faces is not significant (*F*-value = 2.465, uncorrected *p*-value = 0.118).

These results suggest that the strength of the relationship between aesthetics and trustworthiness (Pearson's *r* = 0.676, *p* = < 0.001) are influenced by the age of presented faces, which is whether it is a child or an adult face but not by its gender or ethnicity. Taken together, the findings suggest that, at a general level, when adult raters make inferences about others' aesthetic and trustworthiness, they do not rate people of different gender or ethnicity differently, but they adopt different strategies for adults and children.

**Figure 1:**
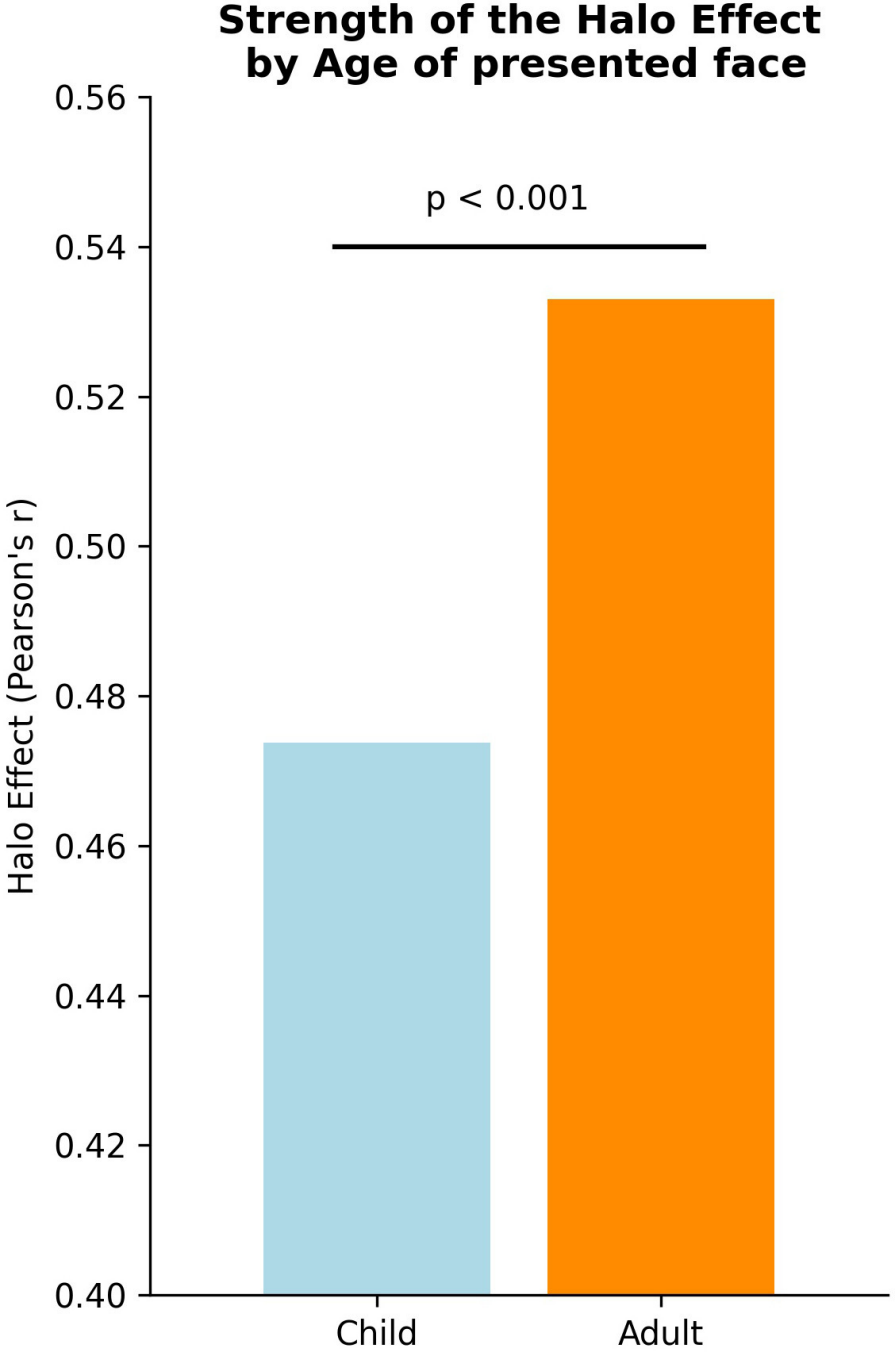
Strength of the halo effect (*pearson-r*) by age.

**Figure 2:**
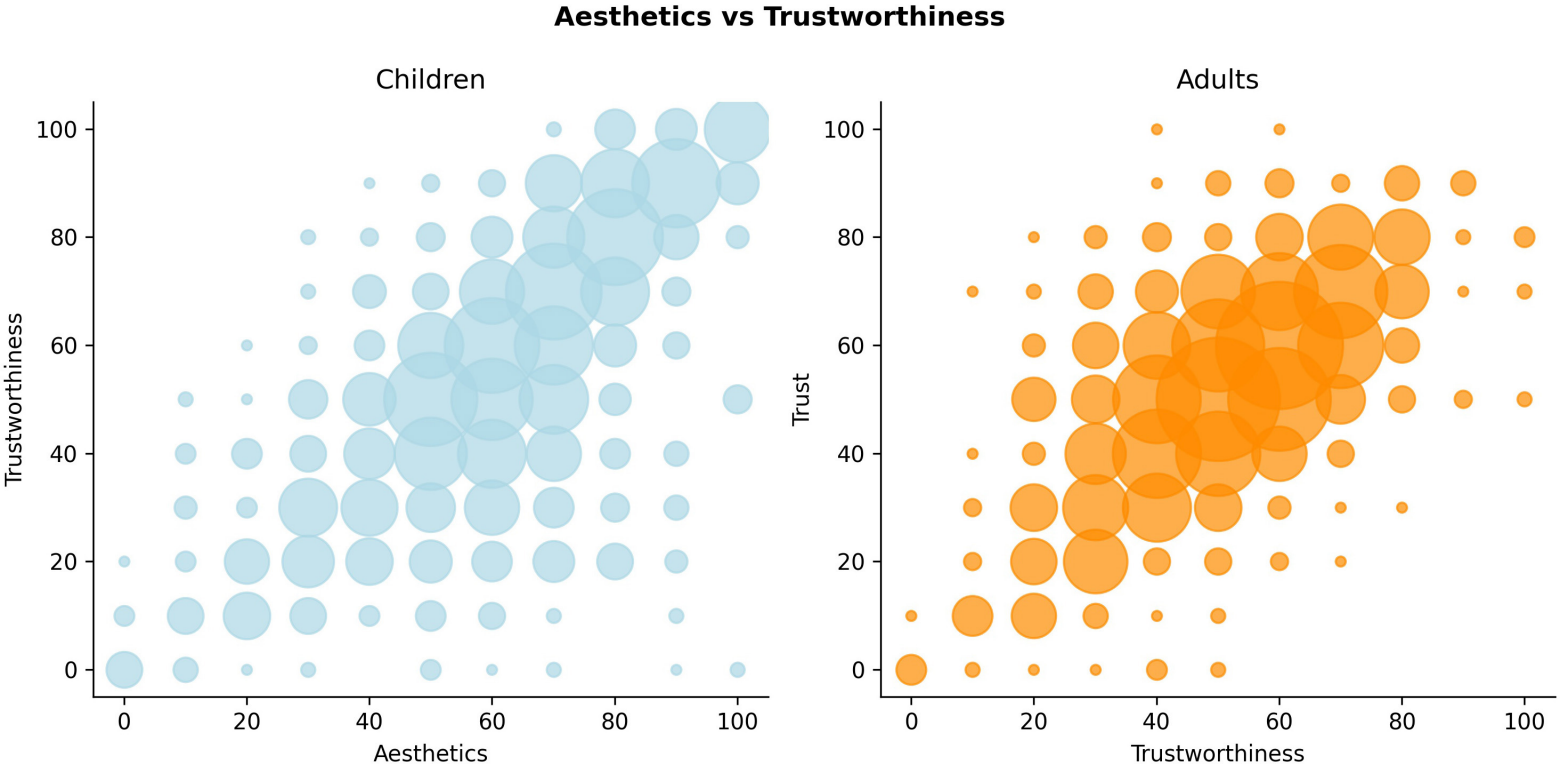
Distribution of aesthetics and trustworthiness judgments by age.

More specifically, the strength of the relationship between aesthetics and trustworthiness is significantly higher (*z*-test *t* = 3.626, *p*-value = 0.000287, Figures 1, 2) for adult (M = 0.53±0.41) than for children faces (M = 0.47±0.46). These results indicate that adults are more likely to estimate the trustworthiness of other adults from their aesthetic appearance, while the estimation is less consistent when it comes to predicting the trustworthiness of children from their appearance.

Additionally, the strength of the relationship between the two variables has been further confirmed using a multiple linear regression analysis, with the formula reported in Equation (1). Results are reported in Table 2.

(1) $$\begin{array}{r} Trustw.=Int.+Aesthetics\times X_{1}+Age\times X_{2}\\ \;+Gender\times X_{3}+Ethnicity\times X_{4} \end{array}$$

A subsequent exploratory analysis revealed that the effect is significantly stronger for Asian participants, as compared to Caucasian participants (*t* = 13.2, uncorrected *p*-value = 9.68·10^-39^). Further exploring the difference between Asian and Caucasian participants, both groups showed no significant differences in the *HE* elicited by younger faces of their same ingroup and outgroup (Asian participants: *t* = −0.67, uncorrected *p*-value = 0.503; Caucasian participants: *t* = −0.935, uncorrected *p*-value = 0.351). Focusing on the behavior of a single ethnic group (e.g., Asians participants), no differences have been found on the correlation of aesthetics and trustworthiness ratings of Asian (ingroup) and Caucasian (outgroup) faces (*t* = −1.551, uncorrected *p*-value = 0.122). On the other hand, the strength of the *HE* —measured as the correlation between aesthetics and trustworthiness ratings—is significantly higher for ingroup (Caucasian) as compared to outgroup (Asian) faces (*t* = 4.026, uncorrected *p*-value = 6.697 ·10^-05^).

**Table 2:** Results of the multiple linear regression used to investigate the strength of the HE and the influence of age, gender, ethnicity, and aesthetic on trustworthiness.

	**Coeff**.	**std. err**	***t***	***P***> |**t**|****	**C.I**.
Intercept	7.4930	1.007	7.445	0.000^*^	[5.519, 9.467]
Aesthetic	0.7797	0.014	55.726	0.000^*^	[0.752, 0.807]
Age	5.1196	0.554	9.243	0.000^*^	[4.034, 6.206]
Gender	0.4762	0.534	0.839	0.372	[−0.570, 1.522]
Ethnicity	−0.2078	0.533	−0.390	0.697	[−1.253, 0.838]

* *P < 0.001*.

### 3.2. Association of SARS-CoV-2 on the Strength of the Halo Effect Over Time

Four (*N* = 4) Levene's tests for equality of variance have been employed to compare the variance of data (aesthetics and trustworthiness) collected before and after the initial diffusion of news about the novel coronavirus (H_2_). The Benjamini–Hochberg procedure, with a false discovery rate of 0.10, is employed to take into account the number of performed tests. Results of the comparison between the variability in aesthetics and trustworthiness judgments toward both adults' and children's faces are reported in Table 3. Results (*q*-values) highlight significant changes in the variability of trustworthiness ratings toward adults' faces before and after the beginning of the COVID-19 pandemic outbreak, but not in aesthetics ratings given to adults' faces, nor to aesthetics or trustworthiness ratings given to children's faces.

**Table 3:** Results of Levene's test of variance for aesthetics and trustworthiness judgments toward adults' and children' faces (*q*-values are evaluated using the Benjamini–Hochberg procedure at a 0.10 false discovery rate).

**Age**	**Variable**	**Statistic**	**Uncorrected *p*-value**	***q*-value**
Adult	Aesthetics	4.633	0.034	0.05
	Trustworthiness	5.557	0.021	0.025
Children	Aesthetics	2.077	0.105	0.1
	Trustworthiness	3.861	0.053	0.075

## 4. Discussion

Based on previous works within the field of the *HE*, we hypothesized that the impact of perceived aesthetic on trustworthiness judgments would depend on the age of presented faces, but not on their gender or ethnicity (H_1_). Results of the analysis of variance show the main effect of the age of presented faces but not of gender or ethnicity, nor of any interaction effect between gender and ethnicity, confirming H_1_. Moreover, our *post-hoc z*-test confirmed that the relationship between aesthetics and trustworthiness is stronger for adults' as compared to children's faces. In light of the results here presented, our analysis supports the specificity of children's faces. In fact, only the age of the presented faces but not the gender or age influenced the strength of the HE in our sample, measured as the Pearson correlation between individuals' aesthetic appearance and perceived trustworthiness. As reported in previous works on the Baby Schema effect (Venturoso et al., 2019), younger faces elicit specific responses in adult viewers. A possible explanation for this may be drawn from the evolutionary perspective. In fact, the cure of the offspring plays a central role in the survival of the species, and therefore adult individuals may be more prone to trust a younger individual even though the perceived aesthetic appearance is low. On the other hand, when looking at adult faces, the evaluation of someone's trustworthiness is largely based on made on the basis of the appearance.

Our exploratory analysis further confirmed the specificity of children's faces. In fact, both Caucasian and Asian participants revealed no significant differences in the strength of the *HE* when exposed to either children of their same ingroup or of their outgroup. While the same can be said for Asian adults looking at Asian and Caucasian adult faces, this does not hold true for for the Caucasians in our pool of participants, who indeed showed significant differences in the strength of the *HE* when exposed to faces of other Caucasians (higher Halo) as compared to adult Asians (lower Halo). This confirms previously published results on both the specificity of children faces, and significant differences in adults' physiological activation (Esposito et al., 2014). While this goes beyond the initial plan of this work and has been in fact not treated as hypothesis confirmation but as exploratory analysis, the general findings here reported about the *HE* are in line with previous works that investigated cross-cultural differences across Asians and Caucasians with different methodologies. Future work should investigate significant differences between the strength of the Halo in Asian and Caucasian participants by properly defining one or more hypotheses and by recruiting an adequate number of participants to verify novel hypotheses with adequate power.

On the subject of the stability of the HE over time (H_2_), the analysis of the variance of data collected before and after the diffusion of news about the novel coronavirus (section 3.2), revealed that adults' faces trustworthiness ratings, but not aesthetics ratings, significantly differ in the data collected before and after the diffusion of news about the novel coronavirus. Differently, no changes are found in the aesthetics and trustworthiness judgments of children's faces. These results are in line with our predictions on the specificity of children's faces. While our results confirm the possibility of modulating the strength of the HE, the current dataset does not allow the study of the qualitative impact of an external event, nor we can claim that changes in the stability are caused exclusively by the current pandemic and public policies. Future studies should address this problem by empirically presenting the external events, using a priming procedure, and measuring the impact over time with a longitudinal and experimental approach.

Despite the strength of the results here presented, there are several limitations worth highlighting. As mentioned earlier, the data collection stage started before and continued during the novel coronavirus pandemic outbreak. To reiterate, significant differences were found in the trustworthiness ratings given to adults faces before and during the pandemic outbreak. Therefore, while our first hypothesis (H_1_) has been empirically verified accordingly to our preregistered plan, we cannot rule out the possibility that the overall world's situation played an indeterminate role in shaping our results, nor that events other than the COVID-19 pandemic outbreak influenced our results. Future works should investigate the stability of the effect under a controlled condition, such as by using a prime. Moreover, while we targeted Asian and Caucasian participants, we have not investigated the influence of participants' ethnicity at a more specific level (e.g., Chinese, Japanese, and Korean). Future studies should focus on a single ethnic group to verify the consistency and generalizability of the results here presented. Additionally, while participants were informed of the scope of the experiment, including the fact that we were specifically interested in aesthetic appearance, participants whose first language is not English may not have a specific counterpart for this concept. Future works should investigate participants' behavior using questions posed in their native language. An additional note has to be placed on the terminology employed in this study. A possible critique is that the experimental setup does not allow to measure aesthetic pleasantness, but liking. While this is a valid critique, participants were informed of the scope of the experiment before enrolling and at the beginning of the experiment. Moreover, our results differ significantly from other works that investigate the relationship between liking and trustworthiness using a similar paradigm [e.g., Todorov et al., 2009, comparison with Study 3 (*N* = 83, ρ = 0.89) *z*-value = 4.816, *p*-value = 0.0002], with the same direction (the strength of the relationship between liking and trustworthiness is higher than the correlation between aesthetic appearance and trustworthiness) reported in other works that compared both the aesthetic appearance and liking with trustworthiness (e.g., Ramos et al., 2016, see Tables 1, 2).

## 5. Conclusion

In this work, we investigated the generalizability and stability over time of the *HE* (esthetic × trustworthiness). Our results show that the strength of the correlation between the perceived aesthetic and trustworthiness of strangers' faces is affected by the age of presented faces, but not by their ethnicity or gender. These results support the body of literature on the specificity of children faces. Moreover, this research serve to add to the limited amount of works that investigated the consistency of the *HE* elicited by aesthetics and trustworthiness across different cultures, and especially in Asian and Caucasian individuals. Additionally, our results show that when a major event that disrupts people's perception of others is presented, such as the SARS-CoV-2 pandemic outbreak, the strength of the association between perceived aesthetics and trustworthiness is less stable for adults' as compared to children's faces. This is, to the best of our knowledge, the first study that examines (i) the effect of gender, age, and ethnicity simultaneously on the strength of the relationship between aesthetics and trustworthiness, as well as the stability of the HE over time when measures that can affect trustworthiness judgments of others (e.g., social distancing) are in place. From a more practical point of view, our results are open to the possibility that external events or actions can affect the relationship between aesthetics and trustworthiness. For example, individuals may use tactics to increase their own perceived trustworthiness or to reduce the perceived trustworthiness of others. We can think of politicians, for example, salesmen, or more in general, activities that require us to interact with a stranger and to evaluate the trustworthiness of a person before approaching or interacting with him or her. Overall, results of our work confirm the generalizability of the HE across cultures, as well as the specificity of children's faces. Additionally, our work provides a first investigation of the stability of the HE over time. Future studies should investigate the effect on more specific ethnic subgroups (e.g., Japanese vs. Chinese), when the stability of the HE is systematically influenced by mean of an experimental paradigm (e.g., priming), and in a period of time where there is a limited influence of external events on judgment toward others' traits.

## Data Availability Statement

The datasets presented in this study can be found in online repositories. The names of the repository/repositories and accession number(s) can be found at: https://doi.org/10.21979/N9/5IIVOM.

## Ethics Statement

The studies involving human participants were reviewed and approved by Institutional Review Board—Nanyang Technological University. The patients/participants provided their written informed consent to participate in this study.

## Author Contributions

GG and GE conceptualized, designed, and conducted the study. AL and PS revised the analytical method. GG drafted the manuscript, while all the authors contributed to the final version of the manuscript. GE supervised the project.

## Conflict of Interest

The authors declare that the research was conducted in the absence of any commercial or financial relationships that could be construed as a potential conflict of interest.
